# The GATA transcription factor GtaC regulates early developmental gene expression dynamics in *Dictyostelium*

**DOI:** 10.1038/ncomms8551

**Published:** 2015-07-06

**Authors:** Balaji Santhanam, Huaqing Cai, Peter N. Devreotes, Gad Shaulsky, Mariko Katoh-Kurasawa

**Affiliations:** 1 Graduate Program in Structural and Computational Biology and Molecular Biophysics, Baylor College of Medicine, Houston, Texas 77030, USA; 2 Department of Molecular and Human Genetics, Baylor College of Medicine, Houston, Texas 77030, USA; 3 Department of Cell Biology, School of Medicine, Johns Hopkins University, Baltimore, Maryland 21205, USA

## Abstract

In many systems, including the social amoeba *Dictyostelium discoideum*, development is often marked by dynamic morphological and transcriptional changes orchestrated by key transcription factors. However, efforts to examine sequential genome-wide changes of gene regulation in developmental processes have been fairly limited. Here we report the developmental regulatory dynamics of GtaC, a GATA-type zinc-finger transcription factor, through the analyses of serial ChIP- and RNA-sequencing data. GtaC is essential for developmental progression, decoding extracellular cAMP pulses during early development and may play a role in mediating cell-type differentiation at later stages. We find that GtaC exhibits temporally distinctive DNA-binding patterns concordant with each developmental stage. We identify direct GtaC targets and observe cotemporaneous GtaC-binding and developmental expression regulation. Our results suggest that GtaC regulates multiple physiological processes as *Dictyostelium* transitions from a group of unicellular amoebae to an integrated multicellular organism.

## Abstract (web summary)

Development involves dynamic transcriptional changes. By serial ChIP- and RNA-sequencing, here, the authors show that GtaC, a GATA type transcription factor, exhibits temporally distinctive DNA binding and regulation of gene expression concordant with the development in the social amoeba *Dictyostelium discoideum*.

## Graphical abstract

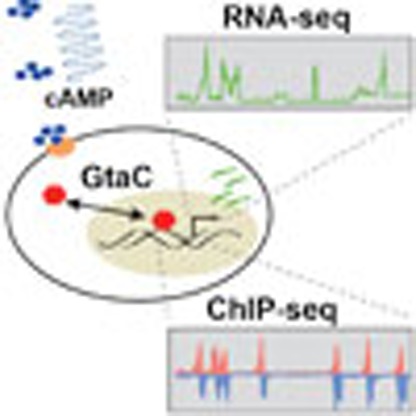

Development involves the sequential execution of molecular events achieved by gene-regulatory networks^1^,^2^ that underlie precise changes in spatiotemporal gene expression patterns^3^,^4^,^5^. Gene-regulatory networks are important biological control systems in which several input and feedback signals are processed to render optimal cellular responses, often by altering overall cellular physiology^1^. Gene expression regulation is mediated through specific interactions between transcription factors (TFs) and *cis*-regulatory DNA elements. Chromatin immunoprecipitation followed by high-throughput sequencing (ChIP-seq) is widely used to assay *in vivo* TF–DNA interactions. This approach provides a genome-wide view of TF-binding preferences, making the discovery of functional *cis*-regulatory DNA elements possible^6^. Genetic perturbation of TFs followed by quantitative measurements of global transcriptional profiles offer another dimension in understanding gene regulation by TFs. Analysing binding preferences of TFs, transcriptome profiling coupled with specific genetic perturbations and finally integrating these binding and transcriptome data aid in providing a comprehensive view of functional regulatory relationships between TFs and their direct targets^7^.

Transcriptional changes accompanying *Dictyostelium* development have been well documented^8^ and efforts have been made to ascertain the developmental roles of several TFs^9^, including GbfA (G-box binding factor A)^10^, STATa (signal transducers and activators of transcription a)^11^, SrfA (MADS-box containing serum response factor A)^12^ and CudA (culmination defective A)^13^. Upon starvation, unicellular *D. discoideum* amoebae initiate a developmental programme and organize into multicellular structures by aggregating in response to 3′-5′-cyclic adenosine monophosphate (cAMP) waves that propagate through the field of cells. After aggregation, cell-type differentiation occurs accompanied by dynamic morphological changes and the developmental process ends in the formation of fruiting bodies consisting of two major cell types—spores and stalks^14^,^15^. *D. purpureum* is an evolutionarily distant dictyostelid species whose developmental morphology is strikingly similar to that of *D. discoideum*. Both species use cAMP as a chemoattractant and their developmental transcriptomes are remarkably conserved^8^,^16^,^17^. One of the conserved transcripts is of *gtaC*, which is abundant in both *D. discoideum* and *D. purpureum* during cAMP-mediated aggregation and encodes a GATA-type TF^8^.

GtaC is a key regulator that decodes extracellular cAMP pulses and establishes downstream signalling pathways, ensuring timely development. During early development, GtaC shuttles between the nucleus and cytoplasm in response to, and out-of-phase with, extracellular cAMP pulses. Cells lacking *gtaC* are defective in aggregation and in morphological and transcriptional progression. Ectopic expression of GtaC fused with green fluorescent protein (GtaC-GFP) in the *gtaC*-null (knockout, KO) strain complements these defects. The expression of a mutated GtaC, in which all four cysteine residues of the zinc-finger domain were substituted with serine residues, does not complement the developmental defects of the KO strain^18^. GtaC may also play a role in cell differentiation during later stages of development^19^. Curiously, *gtaC* transcript abundance increases twice during development. Once during early aggregation and once starting at late aggregation and continuing through the end of development (dictyExpress (http://dictyexpress.biolab.si/)). This observation is broadly consistent with the early and late phenotypes of the *gtaC*-mutant strains^18^,^19^. The possible dependence between the early and late phenotypic manifestations of *gtaC* has not been examined yet.

*Dictyostelium* development is accompanied by sweeping changes in gene expression^8^,^9^, but the regulatory events that mediate these changes are not well understood^20^,^21^. Defects in *gtaC* manifest as an early developmental phenotype^18^, making it an ideal candidate to begin elucidating the *Dictyostelium* developmental gene-regulatory network. In this study, we analyse sequential genomic occupancy data of the wild-type (WT) and cysteine-substituted (CS) forms of GtaC using ChIP-seq to explore the developmental roles of GtaC further. At contemporaneous developmental stages, we also analyse serial transcriptional profiles using RNA sequencing (RNA-seq). By integrating these binding and transcriptome data, we identify GtaC target genes. We find that GtaC targets include developmentally essential TFs and genes involved in cAMP signalling during early development and those relating to cell-type specification at later stages. Our results suggest that GtaC regulates early development by controlling different physiological processes concordant with developmental progression in *Dictyostelium*.

## Results

### Temporal regulation of GtaC–DNA interactions

**Figure 1:**
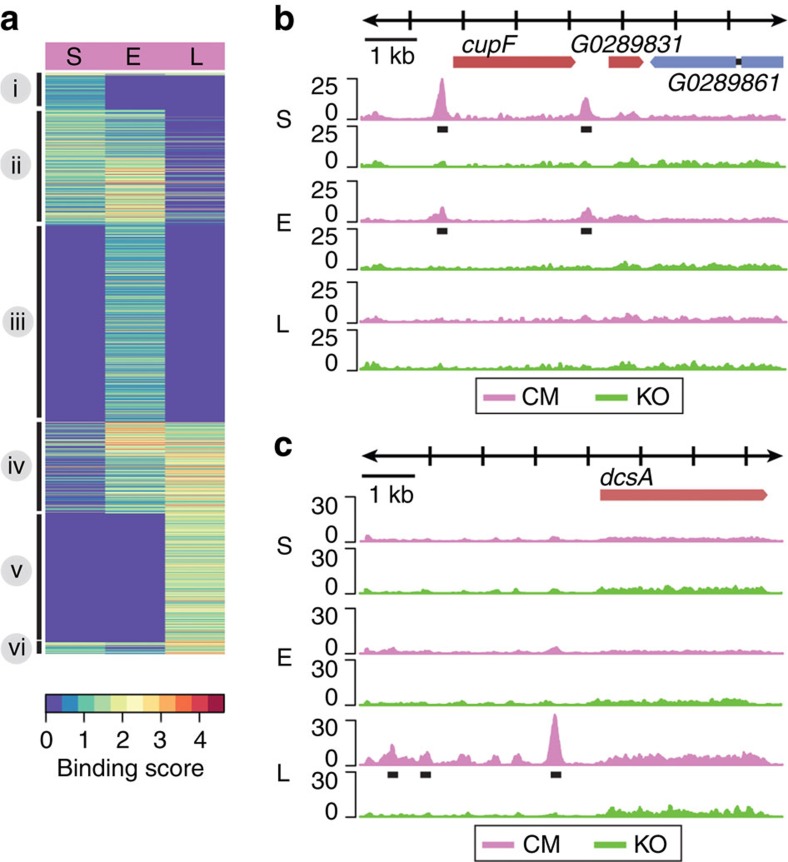
Genome-wide occupancy of GtaC during development. (**a**) Heatmap representation of binding scores (log_2_-transformed, scale indicated) of genes bound by GtaC in their promoter regions during three developmental stages (S—starvation-sensing, E—early aggregation and L—late aggregation; *n*=2,091). Each row represents a gene and each column represents a developmental stage. Rows are ordered on the basis of hierarchical clustering with optimized leaf ordering and grouped (black lines, left) on the basis of temporal patterns of binding events. (**b**,**c**) Representative examples of ChIP-seq patterns (scaled coverage on the *y* axis, DNA length in kilobases on the *x* axis, as indicated at the top). The colours of the tracks indicate the genotypes of the cells (purple: *gtaC*-null complemented with ectopic GtaC-GFP (CM), green: *gtaC*-null GFP (KO)). All the predicted gene models within the genomic region are shown. The exons are coloured depending on the orientation of the gene (red: positive, blue: negative) and introns are shown as black lines. Binding data are shown during starvation-sensing (S), early aggregation (E) and late aggregation around the (B) *cupF* and *DDB_G0289831* loci on chromosome 5, and the (C) *dcsA* locus on chromosome 1. Identified peaks are indicated (black lines).

GtaC is essential for early development in *D. discoideum* and its nuclear localization is regulated by pulsatile cAMP signals during aggregation^18^. To examine the association of GtaC with genomic DNA, we performed ChIP-seq using the CM strain, in which ectopic expression of GtaC-GFP complements the *gtaC*-null mutation, and used the GFP-expressing KO strain as a negative control to exclude potential binding by GFP (Supplementary Fig. 1a, Supplementary Table 1). We developed the cells and collected samples during initial starvation (S), early aggregation (E)—as waves of cAMP are observed— and late aggregation (L)—as the cAMP concentration increases. We identified 2,091 genes whose promoter regions were bound by GtaC (greater than fourfold enrichment in coverage between sample and control ChIP-seq experiments) at any of the three time points (Supplementary Fig. 1b), more than 70% of which were bound at early aggregation (clusters ii, iii and iv in Fig. 1a, Supplementary Table 2). A representative example is shown in Fig. 1b, wherein the upstream regions of the *cupF* and *DDB_G0289831* loci were associated with GtaC during starvation-sensing and early aggregation, but not during late aggregation. We found 462 genes, including *dcsA* (Fig. 1c), which were bound by GtaC only during late aggregation (cluster v in Fig. 1a). In fact, more than 60% of the genes were bound only at one stage (clusters i, iii or v in Fig. 1a, Supplementary Table 2), indicating that GtaC exhibits highly dynamic DNA binding throughout early development. Two central components of the cAMP signalling apparatus—the early adenylyl cyclase gene *acaA* and the early cAMP-receptor gene *carA*, both of which utilize alternative promoters^22^,^23^, were bound by GtaC at all three stages (cluster iv in Fig. 1a). The temporal GtaC-binding patterns were consistent with both the promoter usage and developmental regulation previously observed in these genes (Supplementary Fig. 2)^22^,^23^. Overall, these results support the hypothesis that GtaC mediates cAMP signalling in this system^18^ by regulating the expression of cAMP-related genes.

### GtaC–DNA binding

**Figure 2:**
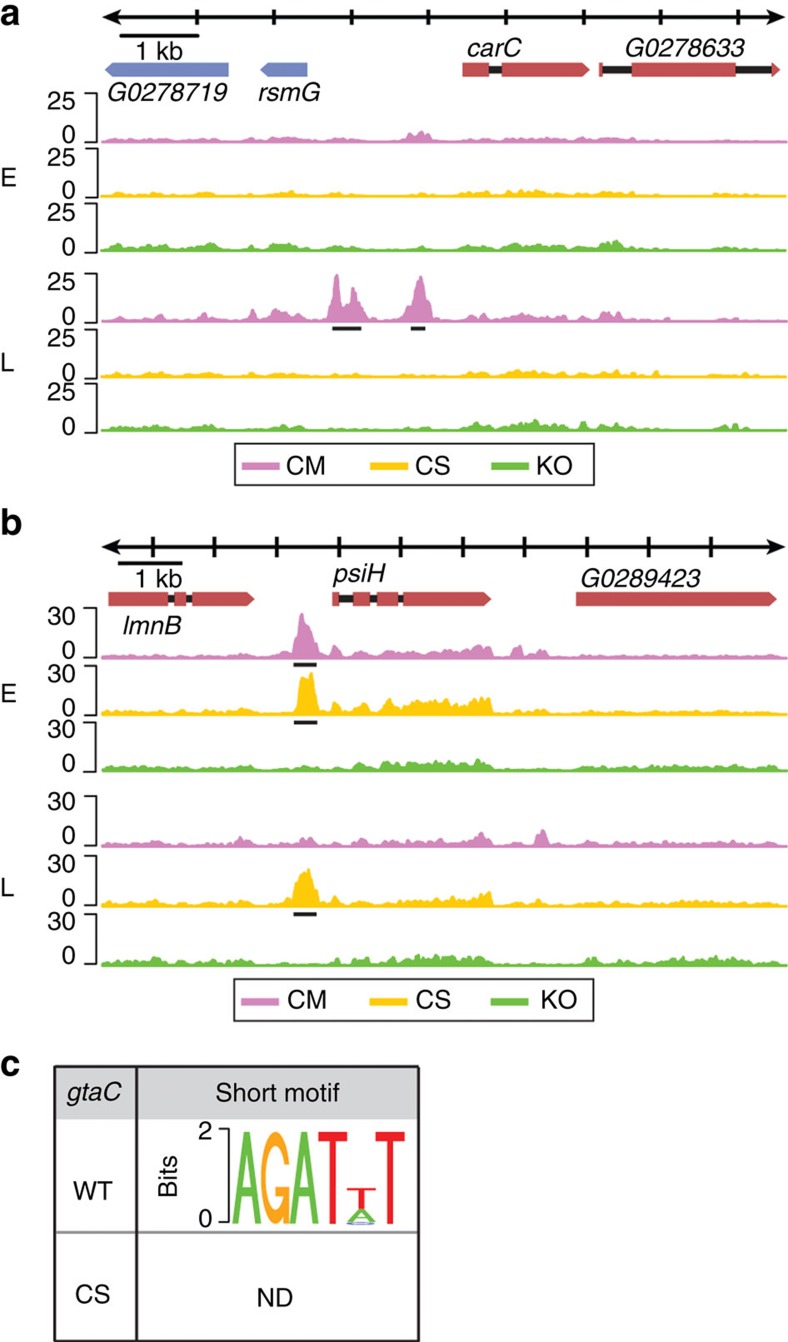
The zinc-finger cysteine residues (C4) affect the DNA-binding specificity of GtaC. Representative examples of ChIP-seq patterns (scaled coverage on the *y* axis, DNA length in kilobases on the *x* axis, as indicated at the top). The colours of the tracks indicate the genotypes of the cells (purple: *gtaC*-null complemented with ectopic GtaC–GFP (CM), yellow: *gtaC*-null ectopically expressing the GtaC^C-S^-GFP (CS), green: *gtaC*-null GFP (KO)). All the predicted gene models within the genomic region are shown. The exons are coloured depending on the orientation of the gene (red: positive, blue: negative) and introns are shown as black lines. Binding data are shown during early aggregation (E) and late aggregation (L) around the (**a**) *carC* locus on chromosome 3 and (**b**) *psiH* locus on chromosome 5. Identified peaks are indicated (black lines). (**c**) We identified enriched motifs using MEME-ChIP^56^ on the 600 most enriched peaks of the WT (top row) and the CS (bottom row) *gtaC* alleles. Information content (bits) is shown on the *y* axis. Motifs identified through DREME (short motif) are shown^57^,^58^. ND indicates that no motifs were detected.

GtaC is a member of the GATA TF family. It contains a Cys_2_–Cys_2_-type IV zinc-finger domain that mediates DNA binding with a zinc ion^24^,^25^. The CS strain carries a mutated form of GtaC in which all four cysteine residues (C4) of the zinc-finger domain were substituted with serine residues (GtaC^C-S^). Expression of GtaC^C-S^-GFP in the KO strain does not complement the defects in aggregation and morphological progression (Supplementary Table 1)^18^. We expected that this mutant form of GtaC would lack DNA binding. To test this hypothesis, we performed ChIP-seq in the CS strain and found that most of the genes bound by GtaC showed no detectable GtaC^C-S^ binding. For example, the upstream regions of the cAMP-receptor gene *carC* were occupied by GtaC, but not by GtaC^C-S^, at late aggregation (Fig. 2a). Surprisingly, we identified 678 genes that showed GtaC^C-S^ binding in their promoter regions (Supplementary Table 2, Supplementary Fig. 1c), most of which (84%) were also bound by GtaC. These results suggest that the zinc-finger C4 mutation changed the propensity but did not completely abolish the ability of GtaC to bind DNA.

To assess the specificity of these interactions, we searched for positional and temporal concordance between GtaC^C-S^- and GtaC-binding events. The binding positions of GtaC^C-S^ and GtaC were remarkably similar in most promoter regions (Supplementary Fig. 3a; top panel), suggesting that GtaC^C-S^- DNA binding represents a mode of normal GtaC–DNA interactions that are independent of the C4 residues in the zinc-finger domain. The extent of overlap between GtaC^C-S^- and GtaC–DNA binding was high during early aggregation (83%) but lower during late aggregation (36%; Supplementary Table 2). In fact, most of the genes bound by both GtaC^C-S^ and GtaC at the earlier time point were bound by only GtaC^C-S^ at the later time point (Supplementary Fig. 3a; bottom panel). A representative example is shown in Fig. 2b, where the upstream region of the *psiH* locus exhibited GtaC-binding at early aggregation but not at late aggregation, whereas GtaC^C-S^ was bound at both times. These results are consistent with the lack of further developmental progression observed in the CS strain and also suggest that GtaC^C-S^ might misregulate its target genes.

To investigate GtaC-binding specificity, we searched for over-represented, short DNA sequences in genomic regions associated with GtaC- or GtaC^C-S^ peaks. We found enrichment for a GATA-like motif only in regions bound by the WT GtaC (Fig. 2c, Supplementary Fig. 3b).

### GtaC regulates developmental gene expression

**Figure 3:**
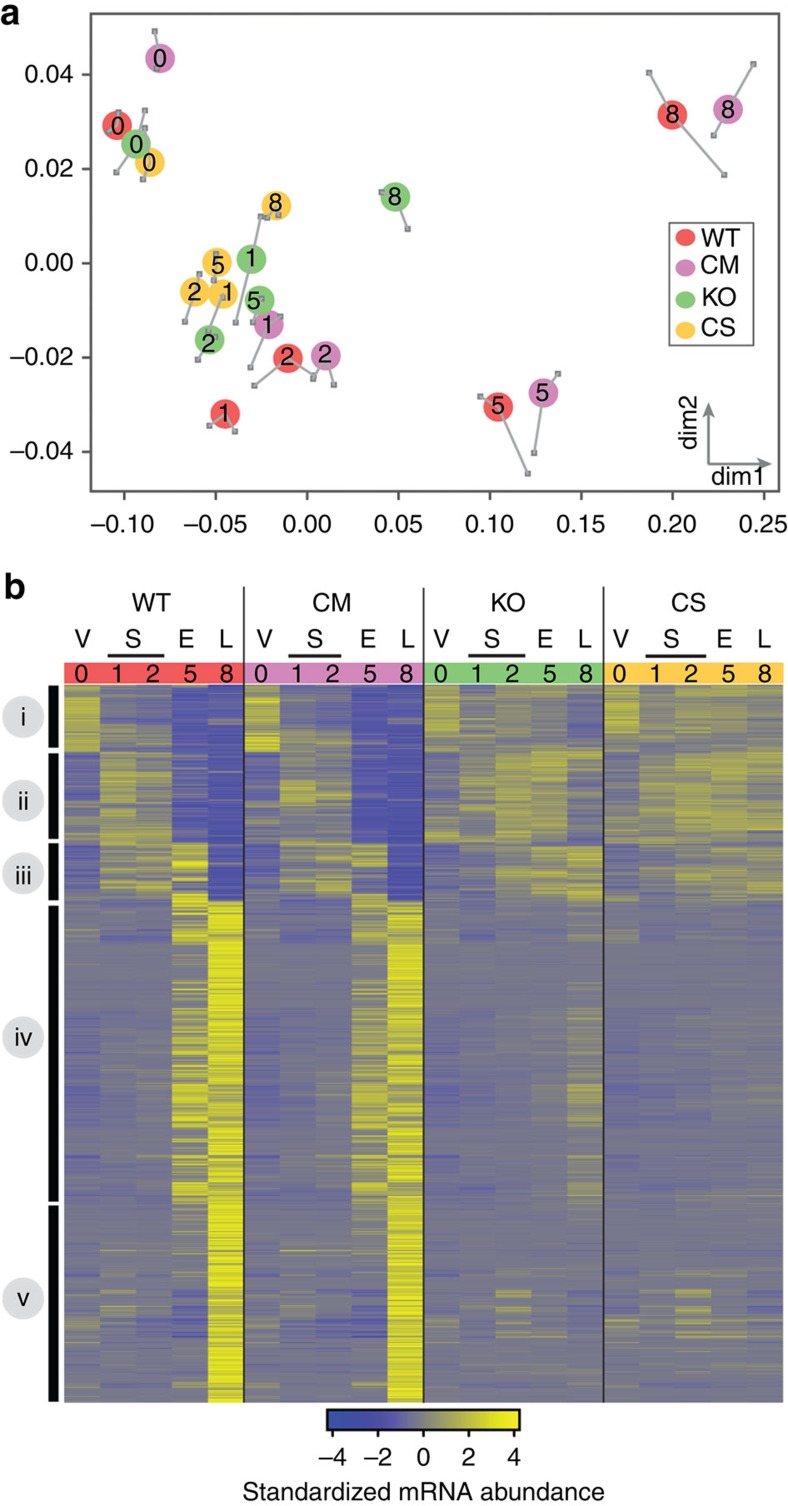
Developmental regulation of GtaC-dependent transcription. (**a**) MDS visualization of distances between transcriptomes. Each coloured circle represents the average transcriptome of two biological replicates (grey squares and whiskers) and numbers inside the circles represent the developmental time (h). Distances on the two-dimensional plane are inversely proportional to the similarity between the transcriptomes. Strains: WT—wild type, CM—*gtaC*-null complemented with ectopic GtaC-GFP, KO—*gtaC*-null, CS—*gtaC*-null ectopically expressing the GtaC^C-S^-GFP. (**b**) Heatmap representation of standardized mRNA abundance of genes (*n*=1,188; scale indicated) that show significant changes between strains that exhibit normal (WT and CM) and aberrant (KO and CS) development. Each row represents a gene and each column represents a transcriptome. Rows are grouped by hierarchical clustering and broad clusters are shown as solid black lines (left). The columns are grouped by strain (coloured panels on top, red: WT, purple: CM, green: KO, yellow: CS) and ordered by time (h, indicated above each column). The time line corresponds to the developmental stage in the WT strain as indicated (V: vegetative growth, S: starvation-sensing, E: early aggregation and L: late aggregation).

To test whether GtaC-binding events were accompanied by gene expression changes, we used our published RNA-seq data of WT, CM and KO strains^18^ and added a few data sets. We first obtained the developmental transcriptome of the CS strain. Since we observed GtaC binding at the late aggregation stage, we also obtained the transcriptomes of all the strains at this stage (Supplementary Data 1, Supplementary Fig. 4). To visualize the differences among the transcriptomes, we used multidimensional scaling (MDS), which provides a low dimensional projection^26^ that best represents the relative distances between individual transcriptomes in their original higher dimensional space (Fig. 3a). We observed that the CS and KO transcriptomes clustered close together. Interestingly, all the CS and KO developmental transcriptomes, other than at 0 h, clustered together with the 1- and 2-h transcriptomes of WT and CM strains at the starvation-sensing stage, indicating a lack of further transcriptional progression. These observations are consistent with the developmental arrest observed in both the KO and CS strains^18^ before the aggregation stage, which normally corresponds to the onset of cAMP signalling. They also indicate that, despite the presence of GtaC^C-S^ in the cells and its ability to bind DNA, the global developmental transcriptomes of the CS strain showed little difference from that of the KO. The WT and CM transcriptomes were similar to one another at each of the time points. They exhibited clear temporal progression such that their transcriptomes at starvation-sensing (1 and 2 h) were closer to each other than to the transcriptomes at early aggregation (5 h) or late aggregation (8 h) stages. Overall, these results suggest that the developmental consequences of carrying various forms of *gtaC* are accompanied and likely caused by overt transcriptional differences between the strains that express them.

We chose to focus on genes that exhibited developmentally regulated changes in their mRNA abundance within each strain over time and between strains at any time point. We found that a majority (∼70%) of the genes in KO and CS strains did not show any significant developmental changes in mRNA abundance, consistent with the lack of transcriptional and morphological progression in these strains (Fig. 3b)^18^. It is therefore likely that most GtaC^C-S^–DNA-binding events have no direct role in the regulation of gene expression. We identified 1,188 genes that were differentially expressed between strains with normal (WT, CM) and aberrant (KO, CS) developmental progression and also developmentally regulated in both WT and CM strains. Using hierarchical clustering, we identified five broad clusters of genes that exhibited coordinated changes in their mRNA abundance over time (Fig. 3b). Genes in clusters i, ii and iii, appeared to be turned on at vegetative growth (V), starvation-sensing (S) or early aggregation (E) stages, respectively, but were turned off by the late aggregation (L) stage in both WT and CM strains (WT and CM in Fig. 3b). This downregulation was compromised in both KO and CS strains (KO and CS in Fig. 3b). Genes in clusters iv and v were upregulated at early (E) and late aggregation (L) stages, respectively, in both WT and CM strains, but not in the KO or CS strains. Some of the transcriptional differences between the WT (or CM) and the KO (or CS) strains, especially at late aggregation, may represent cumulative effects due to the early developmental defect observed in both the KO and CS strains. Notwithstanding, these results suggest that GtaC is a key factor that affects different groups of developmentally regulated genes, influencing both timing and the up/downregulation of their expression.

### Gene-regulatory consequences of GtaC and GtaC^C-S^ binding

**Figure 4:**
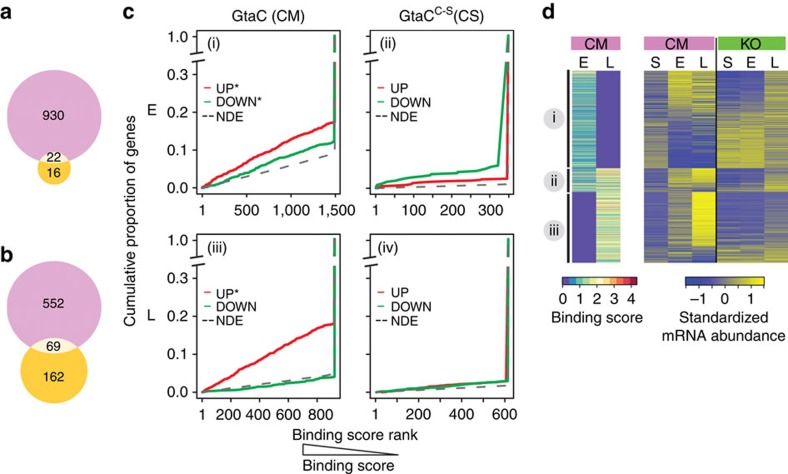
Regulatory consequences of GtaC and GtaC^C-S^ promoter-binding events on gene regulation. (**a**,**b**) Venn diagrams showing the overlap between genes bound by GtaC and differentially expressed between *gtaC*-null cells complemented with ectopic GtaC–GFP (CM) and *gtaC*-null (KO) cells (purple circle) and, genes bound by GtaC^C-S^ and differentially expressed between *gtaC*-null cells ectopically expressing GtaC^C-S^-GFP (CS) and KO cells (yellow circle) during early (**a**) and late (**b**) aggregation. (**c**) Plots showing the cumulative proportions of genes (*y* axis) at a given binding score (ranked; *x* axis) of GtaC or GtaC^C-S^ at early aggregation (E; (i), (ii)) and late aggregation (L; (iii), (iv)). All genes are categorized as upregulated (UP, red lines), downregulated (DOWN, green lines) or NDE (black dashed lines) in CM (first column) or CS (second column) compared with KO cells. The UP and DOWN cumulative distributions are compared with the corresponding NDE distribution using the Kolmogorov–Smirnov test (*P* values<0.001 indicated by * in (i) and (iii)). (**d**) The heatmaps represent GtaC-binding scores (log_2_-transformed; scale indicated, left panel) and standardized mRNA abundance (scale indicated, right panel) of genes bound only by GtaC at early aggregation (E; clusters i–ii) or late aggregation (L; clusters ii–iii). Each row represents a gene and genes were grouped by their binding patterns. Each column represents a strain (coloured panels on top, purple: CM, green: KO) and time point (S: starvation-sensing, E: early aggregation and L: late aggregation).

To explore the regulatory effects of GtaC and GtaC^C-S^ binding, we tested the relationship between binding events on the basis of the ChIP-seq data and transcriptional changes in the RNA-seq data at the corresponding time points. We identified genes bound by GtaC and also differentially expressed between CM and KO strains at the two aggregation stages (E: 952 genes, Fig. 4a; L: 621 genes, Fig. 4b). In addition, we identified genes bound by GtaC^C-S^ and differentially expressed between the CS and KO strains at these two time points (E: 38 genes, Fig. 4a; L: 231 genes, Fig. 4b). We found that a small but significant fraction of these two gene sets overlapped at either time points (22 in Fig. 4a and 69 in Fig. 4b).

To assess these data more quantitatively, we tested whether binding events in the promoters were more likely associated with up- or downregulation of gene expression^7^. We first categorized all genes as upregulated (UP), downregulated (DOWN) or not differentially expressed (NDE) in CM or CS strains compared with the KO strain. We then compared binding score distributions of genes categorized UP or DOWN with those categorized NDE, in each strain (Fig. 4c, Supplementary Table 3). We found that GtaC-binding events were significantly associated with both up- and downregulation of gene expression at early aggregation (Fig. 4c(i)) and only with upregulated genes at the late stage (Fig. 4c(iii)). In contrast, GtaC^C-S^ binding was not associated with up- or downregulation of gene expression at both stages (Fig. 4c(ii,iv)). In fact, of the 245 genes (from Fig. 4a,b) that were bound and differentially expressed in CS compared with KO at either time point, only two genes displayed similar expression levels between CM and CS strains. Taken together, these results suggest that, despite the apparent interaction of GtaC^C-S^ with DNA, the binding events mostly result in faulty regulation of target gene expression.

To further evaluate the regulatory consequences of GtaC binding to GATA-like sequences, we chose genes bound only by GtaC at early or late aggregation and tested whether GtaC binding to the motif was associated with coordinated transcriptional changes in CM but not in KO strains. About half the genes bound by GtaC only at early aggregation showed increased abundance in CM at this time compared with starvation-sensing, and this upregulation was not observed in KO (Fig. 4d, cluster i). The other half, however, was low at early aggregation in CM and remained low until the late stage (Fig. 4d, cluster i). This observation suggests that the low abundance at late aggregation is likely due to indirect effects. On the other hand, most genes bound by GtaC at late aggregation were clearly upregulated at this time in the CM, but not in the KO (Fig. 4d, clusters ii–iii). These results suggest that GtaC binding to GATA-like sequences has a direct role in upregulating gene expression (Fig. 4c(i,iii), Fig. 4d); however, other mechanisms may be required to differentially regulate target genes at different stages.

### Identifying direct targets of GtaC

We defined direct targets of GtaC as genes that were associated with GtaC binding and showed differential mRNA patterns between the CM and KO strains. We used a rank-product (RP) score to integrate these two parameters^7^ such that genes ranked high in both the binding and transcriptome data sets had smaller GtaC–RP scores. We considered the 561 genes with GtaC–RP<0.005 at least in one of the three time points as likely direct targets of GtaC (GtaC targets; Supplementary Data 2). We also tested whether differences in transcript abundance of GtaC targets between CM and WT could be the result of ectopic expression of GtaC-GFP in CM. Comparing the two strains revealed that overall, transcript abundance genome wide, including those of GtaC targets, were remarkably consistent at each of the time points tested (Fig. 3a, Supplementary Fig. 5 (Spearman's correlation (SC)>0.95)). These findings suggest that GtaC-dependent transcriptional regulation in both the CM and WT strains are quite similar and therefore the GtaC-GFP protein is a good representative of the native GtaC function. As a measure of functional correlation between temporal patterns of GtaC binding and gene expression, we calculated their correlation for each gene. More than 60% of the GtaC targets showed similar trends in both (absolute ranked correlation>0.5), indicating that GtaC binding and expression of downstream genes were cotemporaneous and suggesting a causative relationship between GtaC binding and regulated gene expression.

To explore the biological processes regulated by GtaC, we examined gene annotations within the putative direct targets. In a direct comparison with previously defined gene sets, we found the sets of ‘cAMP-responsive'^27^ and ‘cell-type-enriched'^8^ genes to be significantly over-represented among GtaC targets (Supplementary Table 4). These observations are broadly consistent with previous descriptions of the two major functions of GtaC during development: cAMP-mediated signalling^18^ and cell-type specification^19^. Our results further suggest that at least some of these genes are directly regulated by GtaC. The set of genes with known developmental functions (obtained from dictyBase (http://dictybase.org/)) and the set of putative TFs^9^ were also over-represented (Supplementary Table 4), suggesting that the regulatory modules involving GtaC and its direct targets play critical roles in development.

*D. discoideum* and *D. purpureum* are two evolutionarily distant dictyostelid species whose aggregation is mediated by cAMP and, their *gtaC* transcript levels are abundant during the aggregative phases of development^8^,^16^,^17^. Hence, we tested for the enrichment of *D. purpureum* orthologues among GtaC-dependent target genes in *D. discoideum*. Genes bound by GtaC during the aggregative stages were significantly enriched in the set of orthologous genes. Furthermore, a significant proportion of GtaC targets in the set of *D. purpureum* orthologues were coordinately expressed during development across the two species^8^. Given their strikingly similar developmental morphologies and transcriptomes, despite the evolutionary distance between the two species, our results suggest evolutionarily conserved developmental roles for GtaC-dependent regulatory modules in these dictyostelids.

**Figure 5:**
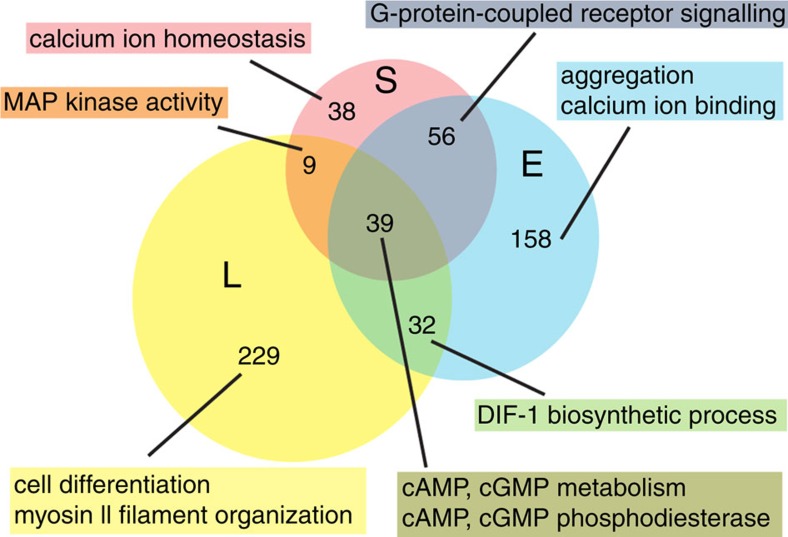
Identifying direct targets of GtaC. A Venn diagram showing the extent of overlap between GtaC target genes (*n*=561) at the three developmental times (S—starvation-sensing, red; E—early aggregation, blue; L—late aggregation, yellow). The number of overlapping genes and the prominent GO terms (*P* values<0.01; Fisher's exact test) enriched in each subset are indicated.

To broaden the association between GtaC targets and *Dictyostelium* developmental processes, we performed an unbiased gene ontology (GO) enrichment analysis and identified enriched GO terms at each time point. The identified GO terms were largely associated with the key developmental processes—‘aggregation' at early aggregation and ‘cell differentiation' at late aggregation, for example, as shown in Fig. 5, Supplementary Data 3. We also discovered annotations related to calcium ion binding and homeostasis, signalling through G-protein-coupled receptors and mitogen activated protein (MAP) kinases as well as cyclic nucleotide metabolism and degradation and differentiation inducing factor 1 (DIF-1) biosynthesis. These results provide novel insights into the role of GtaC in development and shed light on critical GtaC-controlled components underlying the gene-regulatory networks governing multiple physiological processes in *Dictyostelium* development.

## Discussion

In this paper, we showed that GtaC exhibits distinct temporal binding patterns during early development, thus alluding to multiple developmental roles. Such variable binding specificities have been reported in other systems, where they appear to result from various regulatory effectors^28^,^29^. Multicellular development is marked by complex signalling events that alter the physiological states of cells concomitantly with morphological progression^30^,^31^, and these cellular stimuli often act to alter the localization and activity of TFs^32^. Temporally regulated binding events are likely regulated by the combined action of many TFs coupled with changes to the chromatin architecture, ultimately resulting in gene expression changes^2^. In the case of GtaC, its nuclear localization and hence transcriptional activity during early development are dependent on cAMP^18^; however, it is unclear what protein modifications and/or epigenetic effects are responsible for the observed changes in binding patterns.

Here we found that the zinc-finger domain of GtaC was essential for specific binding to the GATA motif. Recent protein-binding microarray experiments revealed that the GtaC DNA-binding domain showed higher-affinity binding to a consensus ‘GATC' sequence, than to ‘GATA' sequences^33^. It should be noted that the strengths of TF binding cannot be inferred from ChIP-seq experiments and the differences in the identified motifs could also be attributed to using the DNA-binding domain in the *in vitro* protein-binding microarray experiments^33^ compared with the full-length GtaC-GFP protein used in our ChIP-seq experiments. Moreover, the sequences bound most avidly by the TF may not necessarily be the most advantageous *cis*-regulatory sequences to modulate gene expression^34^,^35^.

Cysteine substitutions in the GATA zinc-finger domain of GtaC did not affect the ability of the protein to translocate to the nucleus but it seemed to completely inactivate its developmental function^18^. Moreover, while the mutations did not completely abolish its ability to bind DNA, they appear to have inhibited direct interaction with GATA-like sequences. GATA zinc-fingers dimerize and can mediate interactions with both DNA and proteins^25^,^36^ and thus, at least some of the GtaC–DNA interactions could be mediated through other proteins. In addition to cooperative binding, DNA motif preferences can vary depending on the conformational state of TFs when they bind DNA^37^. Another possibility is the existence of additional, uncharacterized DNA-binding domains in GtaC, which remain unaltered in GtaC^C-S^. While it is tempting to speculate that GtaC binds DNA in different modes, more careful analyses are necessary to characterize the existence and functional consequence of GtaC–DNA interactions. However, regardless of the molecular mechanism, GtaC^C-S^ binding appears to have almost no effect on development because ectopic expression of GtaC^C-S^ in the *gtaC*-null strain does not significantly modify the developmental or the transcriptional phenotypes.

We integrated DNA-binding and transcriptome data from the CM and KO strains to identify putative direct targets of GtaC and found good concordance between the data sets. Despite ectopic expression of GtaC-GFP in CM, remarkable similarities between the CM and WT strains, not merely on the basis of gross morphologies, but also at the more detailed resolution of their transcriptional phenotypes suggest that the artificially constructed CM strain is a faithful representation of native GtaC-regulatory function. Temporal patterns of GtaC binding in promoters and expression patterns of the associated genes are largely cotemporaneous, suggesting a causative relationship between TF binding and transcription of targets. Among the GtaC targets, we found enrichment in two broad developmental processes during the early and late times. Our results are consistent with GtaC's role in cAMP-mediated processes during early development^18^. The enrichment of processes relating to cell differentiation during the late time point broadly agrees with the previously described roles of GtaC in cell-type specification^19^. The temporal regulation of GtaC–DNA binding is consistent with this dual role in development. Since our experimental design did not directly test cell-type-specific functions of GtaC, the possibility that the identified late function may be because of cumulative effects of its early roles, however slim, cannot be completely ruled out.

Another interesting observation is the over-representation of co-regulated orthologues between *D. discoideum* and *D. purpureum* among GtaC targets. Given the similarities between the two, our data suggest that some of the GtaC-dependent regulatory modules are conserved between these evolutionarily distant species^17^. Many GtaC targets, such as *acaA* and *carA*, are essential for *D. discoideum* development, reinforcing the notion that GtaC is a critical regulator of development. The functional importance of GtaC targets is also illustrated by the abundance of putative TFs. A number of these TFs, such as GbfA and CudA, also play important roles in *Dictyostelium* development^10^,^13^. The critical role that GtaC has during *D. discoideum* development, the essential functions it regulates and the proposed evolutionary conservation establish GtaC as a significant node in the transcriptional regulatory network that governs *Dictyostelium* development.

Many studies that utilize high-throughput technologies, such as ChIP-seq and RNA-seq, provide a snapshot of the cellular population average^38^,^39^. These data are invaluable to obtain a coarse overview of the molecular events that underlie broad biological processes. However, the dynamics of TF occupancy and its effect on gene regulation often occur with varying timescales and heterogeneity. Therefore, temporal linkage of these data is essential to understand general features of gene networks; however, it is still difficult to resolve with mere static population averages^39^. The dynamics of intracellular processes are often dependent on mechanisms used in processing and responding to time-varying input signals^18^,^39^,^40^,^41^. Our previous findings detailed an oscillatory nuclear localization behaviour of GtaC in response to pulsatile cAMP during the early aggregative phase in development^18^. The *csaA* gene (contact site A), that we identified as a direct GtaC target, is transcribed periodically, with periods similar to that of cAMP pulses^42^ and coinciding with GtaC nuclear accumulation, albeit with a slight delay^18^. Further experimentation, in the presence or absence of pulsatile cAMP, at the single cell level and at finer timescales would be required to test whether this is a general phenomenon of GtaC-dependent transcriptional regulation. Our study provides genome-wide molecular snapshots at different stages of development and it serves as a useful hypothesis generating tool to identify and characterize more such TF–target relationships.

## Methods

### Cell culture and development

We maintained each *D. discoideum* strain at 22 °C in shaking suspension in HL5 medium with the indicated supplements (Supplementary Table 1). To induce synchronized development, we collected mid-log phase cells and washed them twice in potassium phosphate buffer (KK2) or developmental buffer (DB)^18^,^43^. Then, we deposited 3.3 × 10^6^ cells cm^−2^ on nitrocellulose filters that were placed on a buffer-soaked filter paper pad or 5.2 × 10^5^ cells cm^−2^ on DB agar. All strains we used in this paper were described in ref. 18.

### Chromatin immunoprecipitation

ChIP using *Dictyostelium* cells was performed as described in ref. 44 with the following modifications. We used developing CM cells at starvation-sensing (S), early aggregation (E) and late aggregation (L) and on CS cells at the two later time points. As a control for the association of GFP with DNA we performed ChIP on a *gtaC*-null strain ectopically expressing GFP at the corresponding time points (Supplementary Table 1). We harvested cells at each time point, incubated them with 1% paraformaldehyde in KK2 buffer at room temperature for 5 min to crosslink and then added 2.5 M glycine solution to a final concentration of 125 mM to terminate the crosslinking reaction. After washing the cells with KK2, we isolated nuclei by resuspending the cells in a detergent solution (40 mM Tris-HCl pH 7.8, 6 mM MgCl_2_, 40 mM KCl, 0.1 mM EDTA, 5 mM dithiothreitol, 1.5% Sucrose and 0.4% NP40) for 10 min on ice^45^. We then resuspended the nuclei in ice-cold lysis buffer (50 mM Tris-HCl pH 8.0, 150 mM NaCl, 2 mM EDTA, 1% Triton X-100 and 0.05% SDS) containing protease inhibitor cocktail (Roche) and 2 mM phenylmethylsulfonyl fluoride, and sheared the chromatin to 100–300 bp fragments by sonication for nine times at 4-s pulses (amplitude: 45%) using a digital sonicator (S-250D; Branson). After brief centrifugation, we incubated the supernatants for 30 min on ice and centrifuged again to remove the insoluble fraction at lower temperature. The nuclear extracts were pre-cleared with protein G Dynabeads (Invitrogen), which were previously blocked with yeast tRNA, glycogen and BSA. We then incubated the pre-cleared extracts with blocked protein G Dynabeads with (2.4 μg) or without anti-GFP antibodies (clones 7.1 and 13.1 mixture: 11814460001, Roche) on a rotator, overnight at 4 °C. We washed the beads twice with lysis buffer, twice with high-salt buffer (50 mM Tris-HCl pH 8.0, 500 mM NaCl, 2 mM EDTA, 1% Triton X-100 and 0.1% SDS), twice with LiCl-wash buffer (10 mM Tris-HCl pH 8.0, 250 mM LiCl, 1 mM EDTA, 1% NP40, and 1% sodium deoxycholate) and twice with TE buffer, followed by elution with elution buffer (10 mM Tris-HCl pH 7.5, 1 mM EDTA and 1% SDS) twice at 65 °C for 15 min, reverse-crosslinking at 65 °C overnight and purification of the ChIP DNA. We then processed the ChIP DNA fragments to generate multiplexed libraries according to the preparation of barcoded sequencing libraries for the Illumina's Genome Analyzer platform^46^ except that we amplified the multiplexed libraries using AccuPrime Pfx DNA polymerase (Life Technologies) at the last step, and performed Illumina sequencing (Illumina HiSeq).

### ChIP-seq data analysis

The sequencing data were mapped to the *Dictyostelium* reference genome^47^ as described in ref. 46. Briefly, we sequenced the libraries (read length=50 bases) and the resulting FASTQ files were mapped using the alignment tool bowtie^48^ (version 0.12.7) allowing only for single hits (-m 1) and trimming unmapped reads up to 10 bp iteratively by 2 bp. To identify genomic regions preferentially associated with GtaC, we performed peak calling using custom R scripts. Briefly, peak calling was performed on 5-kb genomic regions using partially overlapping (50% overlap), sliding windows of 200 bp. For each window, we calculated the log ratio of the scaled coverage (scaled by number of mapped reads) between sample and control ChIP-seq experiments while permitting a maximum of five mapped reads to share the same genomic coordinates. At each time point, we identified GtaC peaks as genomic regions preferentially associated with GtaC in the sample compared with control (enrichment >4-fold; false discovery rates (FDR)<0.01; read support >50). In the proximity of candidate peaks, we accounted for shifts (up to 200 bp) in the distributions of reads mapping to positive and negative strands^49^ and enforced similar read support on either strand (absolute log ratio<0.5). In addition, we used MACS^50^ to identify peaks (parameters: shiftsize=150, bw (band width)=200, slocal (small local region)=5,000). Only peaks that were commonly identified using both methods were used, and we focused on peaks in intergenic regions for further analyses. We used MEME-ChIP^50^ (motif lengths 4–12 bp; *E*-value threshold: 10^−3^) to search for over-represented motifs on the 600 most enriched GtaC- and GtaC^C-S^ peaks.

To associate peaks with genes, we defined the promoter region of a gene as the region 5′ of the ORF, up to the nearest upstream gene, and assigned all peaks within a promoter region to the gene. We calculated the binding scores of each gene using all peaks in its promoter region on the basis of a scoring scheme adapted from refs 51, 52 with an exponentially decreasing function of its percent distance to the gene start identified on the basis of existing gene models obtained from dictyBase (http://dictybase.org/), weighted by the differential enrichment in GtaC coverage between sample and control ChIP-seq experiments.

### RNA-seq

We collected RNA samples in duplicates from WT, CM, KO and CS strains (Supplementary Table 1). The time points sampled corresponded to the vegetative growth (V; 0 h), starvation-sensing (S; 1–2 h), early aggregation (E; 3–6 h) and late aggregation (L; 7–9 h) stages of WT development. The data sets from WT, CM and KO strains at starvation-sensing, early aggregation and late aggregation were published in ref. 18, and the raw data were deposited under the accession number GSE54866 in the Gene Expression Omnibus. We prepared multiplexed cDNA libraries using these RNA samples as described^18^,^46^ and sequenced them on Illumina HiSeq.

### Transcriptome analyses

The resulting sequences were mapped to the *Dictyostelium* reference genome^46^. Relative distances between the transcriptomes were visualized using classical MDS (R function cmdscale) and using hierarchical clustering with bootstrapping (R package ‘pvclust' version 1.2–2)^53^ with optimized leaf ordering (R package ‘cba' version 0.2–14). We used SC to calculate the distance (*D*=1−SC) and complete linkage as the clustering criterion. The transcriptome analyses yielded data on the steady-state mRNA levels of 12,435 genes, ∼3% of which were not expressed under any of the conditions sampled (Supplementary Data 1). In all cases, the biological replicates were very similar to one another (SC≥0.97).

We performed differential expression (DE) analyses using baySeq^54^ as described in ref. 55. Briefly, we compared the transcriptomes of each genotype and developmental time point to one another and used custom R scripts (baySeq R package version 1.16.0) to perform these analyses. For each comparison, we considered genes with FDR lower than 0.05 and likelihoods greater than 0.9 to be differentially expressed. We identified genes that were developmentally regulated over time in WT and CM cells and differentially expressed between WT and KO, CM and KO, WT and CS, and CM and CS at any time point. Their standardized mRNA abundances were visualized as a heatmap (R function heatmap.2). At each time point we categorized differentially expressed genes as UP or DOWN if their average normalized mRNA abundance was higher or lower, respectively, in CM compared with KO cells. Genes that were differentially expressed between KO and CS cells were also categorized as UP or DOWN if their average normalized mRNA abundances were higher or lower, respectively, in CS compared with KO cells. In DE comparisons between two genotypes, we calculated the DE scores of genes with FDR<0.05 as the log ratio of their average normalized expression on the two genotypes. For all other genes, DE scores for this comparison were set to 0.

### Combining transcriptome and binding data

We identified the number of genes that were bound by GtaC and GtaC^C-S^ and differentially expressed between CM and KO and CS and KO strains, respectively, at both E and L, and visualized the overlap as Venn diagrams (R package ‘Vennerable' version 3.0/r82, R function Venn). To test whether the proportion of overlapping genes identified in our data was different from expected by chance, we randomly sampled the same number of genes from the genome, as observed in our data, at both time points. We counted the number of overlapping genes and obtained an empirical distribution by repeating this procedure a million times. At each time point, we used this distribution to calculate the number of instances that showed a larger overlap than in our data and found that probability of such an event was close to zero. In order to test for regulatory relationships between TF binding in the promoters and mRNA abundance of the downstream genes, we obtained distributions (R package ‘Hmisc' version 3.14-4, R function Ecdf) of GtaC- and GtaC^C-S^-binding scores on genes categorized as UP, DOWN or NDE at each time point in CM and CS cells. We used a nonparametric statistical test (Kolmogorov–Smirnov test; R function ks. test) to compare the binding score distributions of genes that are differentially expressed (UP or DOWN) and those that are not (NDE).

To identify enriched short DNA sequences among developmental-stage-specific gene sets, we first identified genes showing large changes in gene expression in CM compared with KO cells (absolute log-ratio >2) at each of the three time points. Then, we used MEME-ChIP^56^ on the promoter regions of the 600 most enriched GtaC-bound peaks to identify enriched motifs.

We ranked the genes on the basis of the absolute values of their DE scores in comparisons between CM and KO strains (genes with highest values first) at each time point. We also ranked genes on the basis of their GtaC-binding scores such that genes with the high binding scores were ranked first. We computed the RP of genes on the basis of their ranked DE scores and their ranked binding scores at each time point^7^ and genes with lower RPs (GtaC–RP<0.005) were considered to be direct targets of GtaC. We performed Gene Ontology enrichment analyses using custom R scripts (R package ‘topGO' version 2.14.0). The GO annotation files for *D. discoideum* were obtained from dictyBase (http://dictybase.org/).

## Additional information

**Accession codes:** The relevant data sets have been deposited in the Gene Expression Omnibus database of National Center for Biotechnology Information under the accession number GSE63151.

**How to cite this article:** Santhanam, B. *et al*. The GATA transcription factor GtaC regulates early developmental gene expression dynamics in *Dictyostelium*. *Nat. Commun.* 6:7551 doi: 10.1038/ncomms8551 (2015).

## Supplementary Material

Supplementary Information Supplementary Figures 1-5 and Supplementary Tables 1-4

Supplementary Data 1 Transcriptome data and differential expression comparisons. This file contains the transcriptome data and differential expression comparisons. The first column header ‘ddb_g' is the unique identifier as defined by dictyBase (http://dictybase.org) and second column header is the name of the gene. This data included the normalized and raw read counts for each gene on each strain (WT, CM, CS and KO) at 0, 1, 2, 5 and 8 hr of development. The strain information is provided in Table S1. Each RNA-seq run was performed in two biological replicates. Columns showing the raw read counts have a suffix ‘r' and those with the normalized read counts have a suffix ‘n'. The average on both biological replicates of the normalized data has a suffix ‘mavg'. The time points are indicated in the column headers for each. Thus the column header ‘CS_1hr_n1' refers to the normalized expression in the first biological replicate of genes in the CS strain at 1 hour of development (0 hour is considered to be the beginning of starvation). Similarly, ‘CS_1hr_r1' refers to the raw reads counts of genes in this data set. The average of both biological replicates (i.e ‘CS_1hr_n1' and ‘CS_1hr_n2') is under the column header ‘CSmavg.1hr'. Each baySeq comparison performed with two predefined models (DE-differentially expressed and NDE-not differentially expressed) between two strains at the same time point or between time points in each strain produces a total of three outputs: Likelihood (Lik), False Discovery Rate (FDR) and additionally provides directionality of expression difference (greater, lesser or equal). Thus the column headers ‘Lik.DE_2hrWTvs.CS', ‘DE_2hrWTvsCS' and ‘FDR.DE_2hrWTvsCS' refer to the Likelihood, direction of differential expression and the false discovery rate, respectively, in a comparison between the 2 hr WT and CS transcriptomes. Similarly, the column headers ‘Lik.DE_CS0hrvs.1hr' and ‘FDR.DE_CS0hrvs.1hr' refer to the likelihood and false discovery rates, respectively, in a comparison between the 0 hr and 1hr CS transcriptomes.

Supplementary Data 2 Set of putative GtaC target genes. This file contains genes bound by GtaC and the set of putative GtaC target genes (multiple data sheets). Column A refers to the unique identifier as defined by dictyBase (http://dictybase.org) and column B to names of genes and. Columns C-E show the GtaC rank-product scores (calculated as described in the text) at starvation sensing, early aggregation and late aggregation respectively. Columns F and G show GtaCC-S rank-product scores at early and late aggregation. The differential expression scores (calculated as described in text) for comparisons between CM and KO at all three times are in columns H-J, respectively and those for comparisons between CS and KO at early and late aggregation in columns K and L, respectively. GtaC binding scores (calculated as described in text) at all three time points are in columns M-O respectively and GtaCC-S binding scores at the latter two time points in columns P and Q respectively.

Supplementary Data 3 Gene ontology (GO) analyses results for GtaC-targets by time point. This files includes gene ontology (GO) analyses results for GtaC-targets by time point (multiple data sheets). Each data sheet contains results on gene sets at starvation sensing (S), early aggregation (E), late aggregation (L), S & E, S & L, E & L and S & E & L, respectively. The unique GO identifier (‘GO.ID'), a short description of the GO term (‘Term'), the number of genes in the genome with that annotation (‘Annotated'), the number of genes in the chosen gene set with the same annotation (‘Significant'), the GO term category (‘GO category') along with all the relevant statistical parameters (‘p-value' and ‘Fold enrichment') are shown for each gene.
