# Supplementary material for: The GATA transcription factor GtaC regulates early developmental gene expression dynamics in Dictyostelium
Source: Nat Commun. 2015 Jul 6;6:7551. doi: 10.1038/ncomms8551 (PMC4506546; doi:10.1038/ncomms8551)
Supplement: Supplementary Information — Supplementary Figures 1-5 and Supplementary Tables 1-4 [file ncomms8551-s1.pdf]

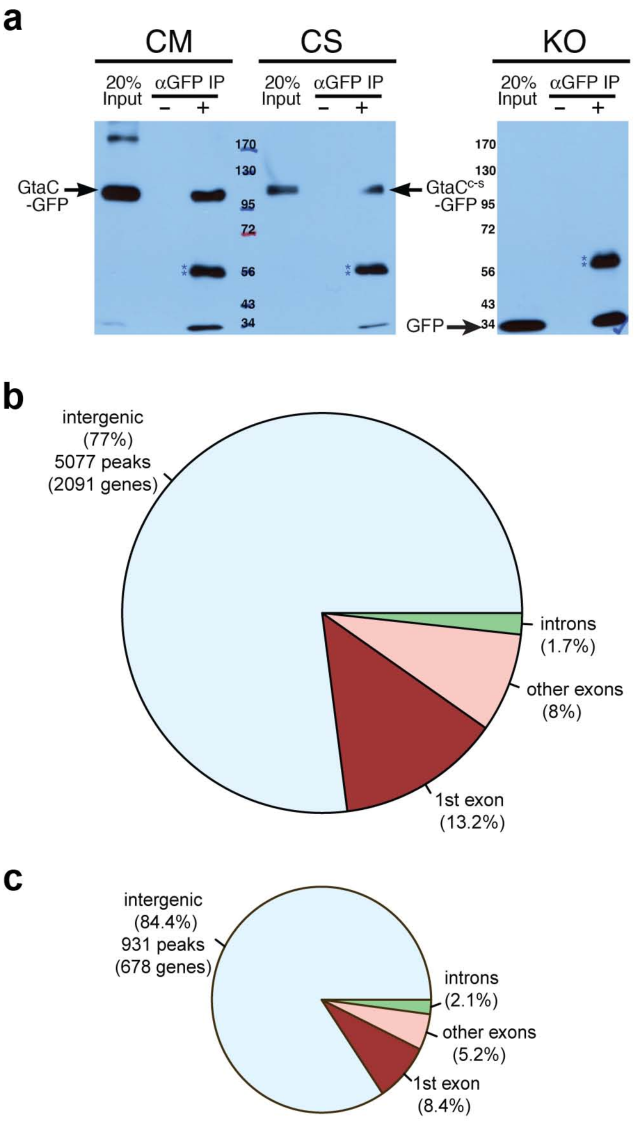


**Supplementary Figure 1: GtaC ChIP and genomic distribution of GtaC-binding events. (a)** ChIP experiments were performed using nuclear extracts (Input) from CM, CS, KO cells with or without GFP antibodies. Immunoprecipitates were visualized by Western blot analysis with another anti-GFP antibody (B-2; sc-9996, Santa Cruz Biotechnology) before the process of libraries preparation for ChIP-seq. Arrows indicate GtaC-GFP and GtaC$^{C-S}$-GFP bands and asterisks represent antibodies bands. **(b-c)** Pie-charts show the percentage of peaks that fall within intergenic, exonic and intronic regions. The distributions reflect GtaC-peaks (b) (n = 7,983) on the CM genome at starvation sensing, early aggregation and late aggregation and GtaC$^{C-S}$-peaks (c) (n = 1,321) on the CS genome at early aggregation and late aggregation.

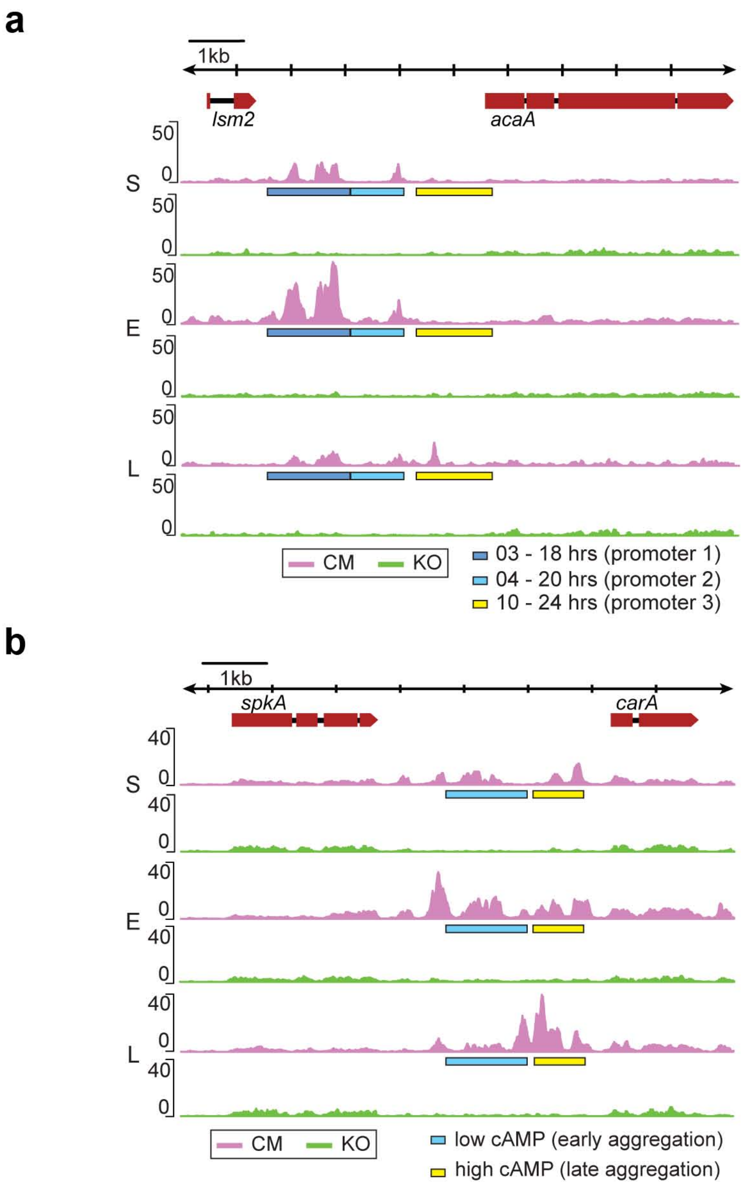


**Supplementary Figure 2: Temporal patterns of GtaC-DNA binding in the promoters of *acaA* and *carA*.** Representative examples of ChIP-seq patterns (scaled coverage on the Y-axis, DNA length in kilobases on the X-axis, as indicated at the top). The colors of the tracks indicate the genotypes of the cells (purple: *gtaC*-null complemented with ectopic wild-type GtaC-GFP (CM), green: *gtaC*-null GFP (KO)). All the predicted gene models within the genomic region are shown. The exons are shown as red boxes and the introns are shown as black lines. Binding data are shown during starvation sensing (S), early aggregation (E) and late aggregation around the (a) *acaA* locus on chromosome 3 and (b) *carA* locus on chromosome 2. The promoter usage and timing of their activity (a) *acaA* and (b) *carA* are indicated as colored boxes below each track and the legends are indicated below the charts.

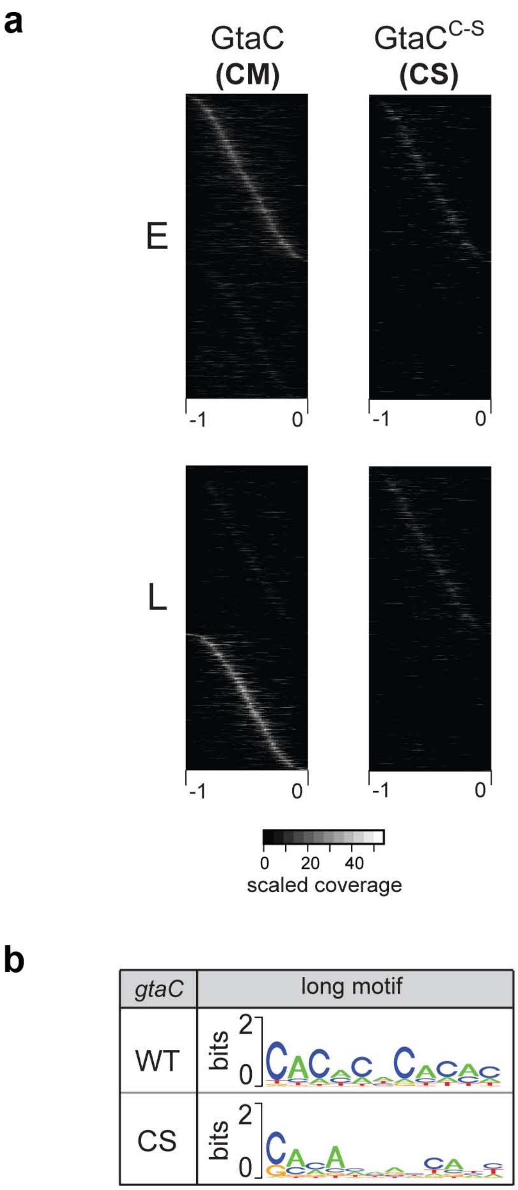


**Supplementary Figure 3: Positional concordance and sequence preferences of GtaC- and GtaC$^{C\text{-}S}$-DNA interactions. (a)** Heatmaps show coverage (scale indicated) of GtaC (CM, left) or GtaC$^{C\text{-}S}$ (CS, right) at early aggregation (E, top panel) and late aggregation (L, bottom panel) in promoter regions of genes that are bound by GtaC at E or L. Unique promoter regions longer than 1 kb are depicted (n=1,006) and promoter lengths are rescaled to unity with 0 indicating the start of the ORF and -1, the end of the promoter region (based on dictyBase gene models (http://dictybase.org/)). Strains: CM – *gtaC*-null complemented with ectopic wild-type GtaC-GFP, CS – *gtaC*-null expressing ectopic cysteine-substituted GtaC$^{C\text{-}S}$-GFP. **(b)** We identified enriched motifs using MEME-ChIP on the 600 most enriched peaks of the wild-type (WT) and the cysteine-substituted (CS) *gtaC* alleles. Information content (bits) is shown on the Y-axis. Motifs identified through MEME (long motif) are shown.

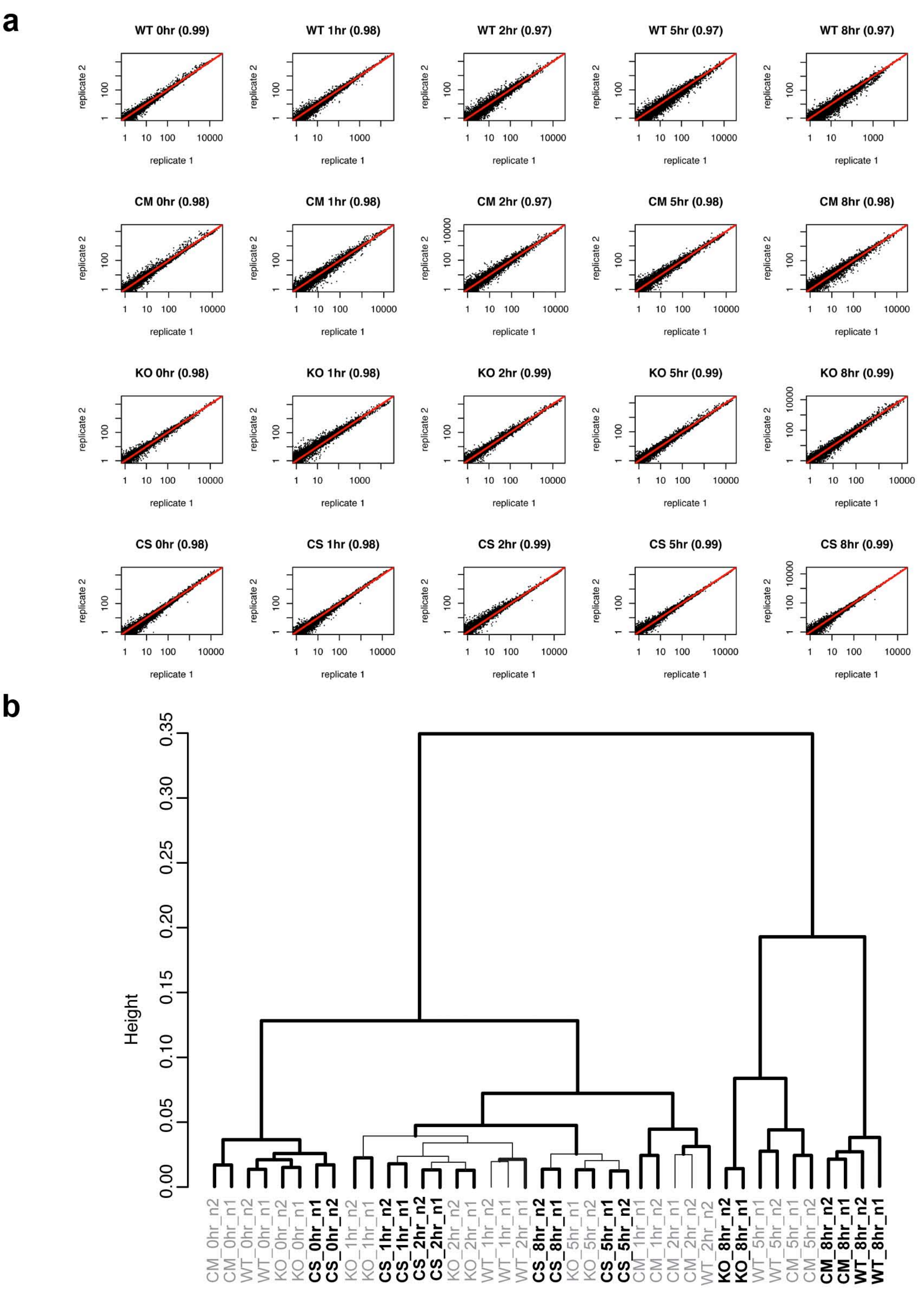


**Supplementary Figure 4: Replicate similarity and clustering of developmental transcriptomes. (a)** The scatter plots show comparisons between standardized mRNA abundances in two biological replicates (X-axis: replicate 1, Y-axis: replicate 2; log scale) of all the genes. Each dot (in black) represents a gene and the red line represents the y=x line. The strain, time-point (in hours) and Spearman's correlation values are indicated for each comparison. Strains: WT – wild-type, CM – *gtaC*-null complemented with ectopic wild-type GtaC-GFP, KO – *gtaC*-null, CS – *gtaC*-null expressing ectopic cysteine-substituted GtaC$^{C\text{-}S}$-GFP. **(b)** Hierarchical clustering with multiscale bootstrap resampling (R package pvclust of normalized mRNA abundances from WT, CM, KO and CS cells at 0, 1, 2, 5, 8 hours of development. Branches with bootstrap-probability less than 0.9 are shown in thinner lines. Distance between two transcriptomes were calculated as D = 1-SC (SC: Spearman's correlation). Transcriptomes obtained for this study are in bold.

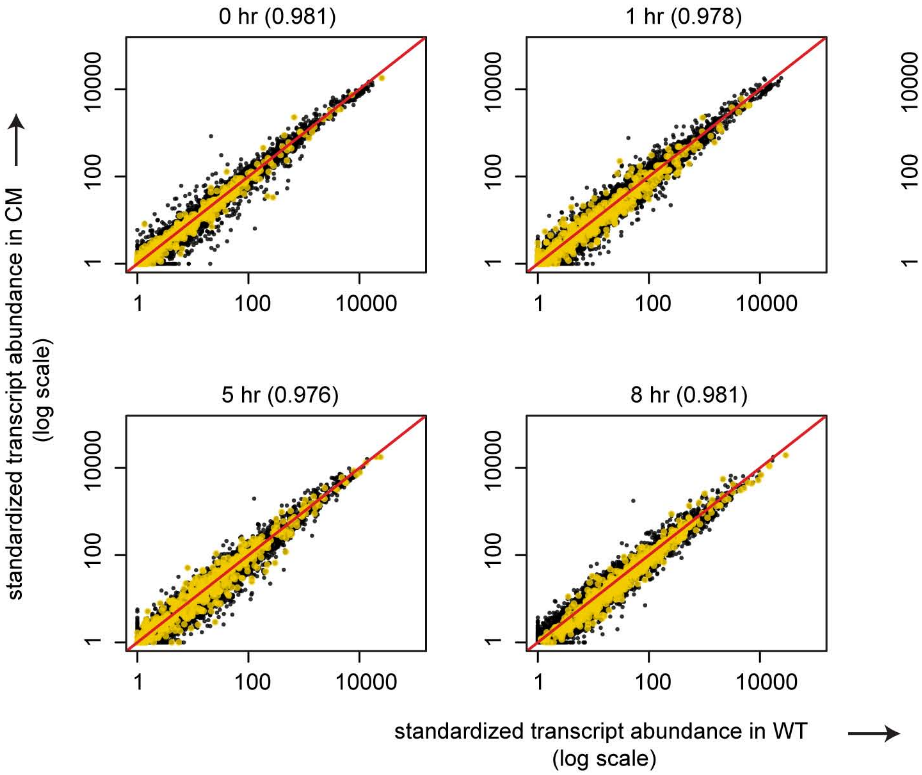


**Supplementary Figure 5: Comparison of transcript abundances between WT and CM strains.** Scatterplots show comparisons between averaged (of two biological replicates) standardized mRNA abundances between WT (X-axis; log scale) and CM strains (Y-axis; log scale) of all genes (black dots) in the transcriptome. Genes identified to be putative GtaC targets are shown in yellow and the red line represents the y=x line. The time point (in hours) and Spearman's correlation values (including the entire transcriptome) are indicated for each comparison. Strains: WT – wild-type, CM – *gtaC*-null complemented with ectopic wild-type GtaC-GFP.

## **Supplementary Table 1:** *D. discoideum* strains used

| Strain Name | Strain Description | Strain Summary | Phenotype | Parental strain | Antibiotic resistance |
|---|---|---|---|---|---|
| WT | AX2 | wild-type | wild-type | AX2 | NONE |
| KO | *gtaC*⁻ or *gtaC*⁻/[act15]:GFP | *gtaC* null | aggregation-defective | AX2 or *gtaC*⁻ | BlasticidinS or G418 |
| CM | *gtaC*⁻/[act15]:GtaC:GFP | complemented mutant | wild-type | *gtaC*⁻ | G418 |
| CS | *gtaC*⁻/[act15]:GtaC$^{C\text{-}S}$:GFP | cysteine substituted GtaC | aggregation-defective | *gtaC*⁻ | G418 |

## **Supplementary Table 2:** The number of genes bound by GtaC and $GtaC^{C\text{-}S}$

| *gtaC* | Time point | Total number of genes | Number of genes bound only at one time point | Overlap with GtaC binding (S) | Overlap with GtaC binding (E) | Overlap with GtaC binding (L) | Overlap with $GtaC^{C\text{-}S}$ binding (E) | Overlap with $GtaC^{C\text{-}S}$ binding (L) |
|---|---|---|---|---|---|---|---|---|
| WT | GtaC binding (S) | 735 | 123 | – | | | | |
| WT | GtaC binding (E) | 1494 | 709 | 600 | – | | | |
| WT | GtaC binding (L) | 922 | 462 | 275 | 448 | – | | |
| CS | $GtaC^{C\text{-}S}$ binding (E) | 348 | 63 | 285 | 288 (*83%) | 142 | – | |
| CS | $GtaC^{C\text{-}S}$ binding (L) | 615 | 330 | 374 | 473 | 219 (*36%) | 285 | – |

* The percentage of overlapping genes with GtaC binding genes in the population of $GtaC^{C\text{-}S}$ binding genes.

**Supplementary Table 3:** Comparisons between binding scores and differential expression categories

| *gtaC* | Differential expression comparison | Time point | Comparison | Kolmogorov-Smirnov test |
|---|---|---|---|---|
| GtaC | CM vs. KO | starvation sensing (S) | UP vs. NDE<br>DOWN vs. NDE | D = 0.1322, p-value = 9.388e-08<br>D = 0.006, p-value = 1 |
| GtaC | CM vs. KO | early aggregation (E) | UP vs. NDE<br>DOWN vs. NDE | D = 0.0999, p-value = 1.797e-13<br>D = 0.0445, p-value = 0.0003211 |
| GtaC | CM vs. KO | late aggregation (L) | UP vs. NDE<br>DOWN vs. NDE | D = 0.1419, p-value < 2.2e-16<br>D = 0.0055, p-value = 1 |
| $GtaC^{C-S}$ | CS vs. KO | early aggregation (E) | UP vs. NDE<br>DOWN vs. NDE | D = 0.0373, p-value = 0.8967<br>D = 0.1241, p-value = 0.02658 |
| $GtaC^{C-S}$ | CS vs.KO | late aggregation (L) | UP vs. NDE<br>DOWN vs. NDE | D = 0.0286, p-value = 0.3738<br>D = 0.0306, p-value = 0.1933 |

**Supplementary Table 4:** Enrichment of pre-defined gene sets in GtaC-targets

| Functional category | Number of genes in genome | Number of genes in GtaC-targets | Fisher's exact test *p*-value | Fisher's exact test Enrichment (odds-ratio) |
|---|---|---|---|---|
| cAMP-responsive | 62 | **23** | 8.30E-16 | 12.96 |
| cell-type enriched | 1332 | **126** | 2.20E-16 | 2.56 |
| developmental mutants | 613 | **70** | 4.00E-13 | 2.97 |
| transcription factors | 263 | **27** | 5.70E-05 | 2.49 |

total number of genes = 12435

**putative GtaC-targets = 561**

cAMP responsive genes (breakdown)

| dictyBase Gene ID | Gene name |
|---|---|
| *DDB_G0281545* | *acaA* |
| *DDB_G0273397* | *carA-1* |
| *DDB_G0276759* | *cbpA* |
| *DDB_G0283613* | *cbpC* |
| *DDB_G0283153* | *cbpD1* |
| *DDB_G0283083* | *cbpD2* |
| *DDB_G0283609* | *cbpG* |
| *DDB_G0288245* | *cigA* |
| *DDB_G0283149* | *cotD* |
| *DDB_G0289073* | *csaA* |
| *DDB_G0269124* | *dcsA* |
| *DDB_G0270190* | *DDB_G0270190* |
| *DDB_G0273079* | *DDB_G0273079* |
| *DDB_G0280557* | *DDB_G0280557* |
| *DDB_G0286025* | *DDB_G0286025* |
| *DDB_G0291253* | *dia2* |
| *DDB_G0276267* | *gpaB* |
| *DDB_G0291998* | *naglu* |
| *DDB_G0280053* | *osbA* |
| *DDB_G0285995* | *pdsA* |
| *DDB_G0288919* | *psiI* |
| *DDB_G0284759* | *psiM* |
| *DDB_G0284331* | *regA* |
